# Designing and evaluating dose-escalation studies made easy: The MoDEsT web app

**DOI:** 10.1177/1740774519890146

**Published:** 2019-12-19

**Authors:** Philip Pallmann, Fang Wan, Adrian P Mander, Graham M Wheeler, Christina Yap, Sally Clive, Lisa V Hampson, Thomas Jaki

**Affiliations:** 1 Centre for Trials Research, College of Biomedical & Life Sciences, Cardiff University, Cardiff, UK; 2 Department of Mathematics & Statistics, Lancaster University, Lancaster, UK; 3 MRC Biostatistics Unit, University of Cambridge, Cambridge, UK; 4 Cancer Research UK & UCL Cancer Trials Centre, University College London, London, UK; 5 Cancer Research UK Clinical Trials Unit, University of Birmingham, Birmingham, UK; 6 Edinburgh Cancer Centre, Western General Hospital, Edinburgh, UK; 7 Statistical Methodology, Novartis Pharma AG, Basel, Switzerland

**Keywords:** Phase I clinical trial, dose-finding study, logistic model, Bayesian statistics, graphical user interface, shiny app, R software

## Abstract

**Background/aims:**

Dose-escalation studies are essential in the early stages of developing novel treatments, when the aim is to find a safe dose for administration in humans. Despite their great importance, many dose-escalation studies use study designs based on heuristic algorithms with well-documented drawbacks. Bayesian decision procedures provide a design alternative that is conceptually simple and methodologically sound, but very rarely used in practice, at least in part due to their perceived statistical complexity. There are currently very few easily accessible software implementations that would facilitate their application.

**Methods:**

We have created MoDEsT, a free and easy-to-use web application for designing and conducting single-agent dose-escalation studies with a binary toxicity endpoint, where the objective is to estimate the maximum tolerated dose. MoDEsT uses a well-established Bayesian decision procedure based on logistic regression. The software has a user-friendly point-and-click interface, makes changes visible in real time, and automatically generates a range of graphs, tables, and reports. It is aimed at clinicians as well as statisticians with limited expertise in model-based dose-escalation designs, and does not require any statistical programming skills to evaluate the operating characteristics of, or implement, the Bayesian dose-escalation design.

**Results:**

MoDEsT comes in two parts: a ‘Design’ module to explore design options and simulate their operating characteristics, and a ‘Conduct’ module to guide the dose-finding process throughout the study. We illustrate the practical use of both modules with data from a real phase I study in terminal cancer.

**Conclusion:**

Enabling both methodologists and clinicians to understand and apply model-based study designs with ease is a key factor towards their routine use in early-phase studies. We hope that MoDEsT will enable incorporation of Bayesian decision procedures for dose escalation at the earliest stage of clinical trial design, thus increasing their use in early-phase trials.

## Introduction

The primary aim of many phase I dose-escalation studies is to estimate the maximum tolerated dose of a novel drug or treatment. In practice, this often means identifying a dose for which the probability of a patient developing a dose-limiting toxicity is close to a prespecified target toxicity level, typically between 0.20 and 0.33 in cancer trials. Patients enter the study in cohorts of one or more (usually three), and for every new cohort a decision is made whether to stay at the current dose level, escalate or de-escalate the dose, or stop the study entirely. A statistical study design informs and guides this process, but the ultimate decision will always be based on clinical judgement.

It is good practice that early-phase dose-escalation designs use a well-fitting statistical model to synthesise all available information (from prior knowledge and accumulating patient data) and deduce a recommendation for how to proceed with the study.^1,2^ The first model-based designs were developed in the 1990s, most prominently the continual reassessment method,^3^ and also Bayesian decision procedures,^4,5^ which are closely related to one another.^6^

The alternative to model-based designs are methods that rely on largely heuristic rules or algorithms, such as the 3+3 design. The only real virtue of these algorithms is their simplicity (at least when nothing unforeseen happens), but on the downside they are less likely to identify the correct maximum tolerated dose than model-based designs, require on average more patients to reach a dose recommendation, have less flexibility to accommodate deviations from the prespecified dose-escalation procedure, and lack any theoretical foundation.^7–10^ Despite these clear drawbacks, rule-based designs are still in wide use, while the uptake of model-based designs remains slow, especially in the public sector.^11–13^ Unfortunately, the latter appear to many as a black box requiring specialist statistical input – which they are not.^14^ There is also a misconception that they are overly complicated because they require more planning than rule-based designs, such as the choice of a prior probability distribution (or ‘prior’ for short).

In this article, we introduce MoDEsT (*Mo*del-based *D*ose *Es*calation *T*rials), a free and easy-to-use web tool for designing and conducting single-agent dose-escalation studies guided by a Bayesian decision procedure to estimate the maximum tolerated dose.^15^ This method is conceptually straightforward and statistically sound: it uses logistic regression to model the relationship between dose and risk of toxicity and allows the investigator to specify prior distributions for dose-toxicity model parameters through the means of ‘pseudo-observations’. These pseudo-observations should represent our best guesses, prior to the start of the study, at the toxicity outcomes that would be recorded if hypothetical (i.e. pseudo-)patients were administered certain doses of the compound. Usually, priors are specified by stipulating pseudo-observations for the lowest and highest doses available for administration during the future study. Prior specification can be informed by a scientific understanding of the drug’s anticipated mechanism of action. Alternatively, the pseudo-observations can be set so as to ensure the procedure has favourable operating characteristics (so-called ‘operational priors’).

Onerous tasks that are currently a barrier to the use of model-based designs, like setting a prior, become easy with MoDEsT: it allows trying out different priors and immediately visualises the consequences in terms of the operating characteristics (e.g. probability of identifying the correct maximum tolerated dose, expected number of patients required) of the procedure. This provides users with insight as to how dose recommendations come about, thus demystifying the model-based design.

In contrast to most other software for study design, MoDEsT is specifically aimed at both clinical trialists and statisticians with no previous experience of model-based dose escalation who would default to the 3+3 design for simplicity, although we believe it is also useful for statistical experts who already have a thorough understanding of model-based dose escalation. The intuitive point-and-click interface of MoDEsT encourages users to explore a variety of design options and allows them to watch changes become effective in real time and get a feel for the design’s performance in different clinically relevant scenarios. It facilitates the consideration and inclusion of efficient model-based dose escalation at the earliest stage of clinical trial design, which should always be a collaborative effort between clinical and statistical experts.

MoDEsT was written in the R^16^ programming language and using the extension package shiny,^17^ which provides a framework for building interactive web applications. shiny is steadily gaining popularity in the context of methods for early-phase dose finding. Recent years have seen the development of shiny apps to

- Design and run dose-escalation studies using the continual reassessment method,^18^
- Design dual-agent dose-escalation studies,^19^
- Compare the performances of various model- and rule-based designs,^20,21^
- Simulate the highest achievable (i.e. optimal benchmark) accuracy when selecting the maximum tolerated dose.^22^

There are also a number of graphical user interfaces for dose-finding methods that are not based on shiny, such as ‘NextGen-DF’ (now called ‘U-Design’),^23^‘Web-EWOC’,^24^ a plethora of tools provided by the MD Anderson Cancer Center (https://biostatistics.mdanderson.org/SoftwareDownload), and commercial packages such as EAST Escalate (https://www.cytel.com/software/east) and FACTS (https://www.berryconsultants.com/software). To the best of our knowledge, none of them holds any functionality for the method outlined in this paper. The only software implementation of this method that we are aware of is ‘Bayesian ADEPT’,^25,26^ which has been defunct for several years. Further distinct strengths of MoDEsT are:

1. It runs under any operating system;
2. It does not require any software package to be installed;
3. Its point-and-click interface means no specialist software or programming skills are required;
4. It automatically generates PDF reports;
5. It is free to use.

## Methods

### Bayesian decision procedure

The Bayesian decision procedure implemented in MoDEsT is made up of four main components: (1) a logistic regression model, (2) prior information about the dose-toxicity relationship, (3) a gain function, and (4) a set of rules for (de-)escalating the dose and stopping the study. We describe each component briefly below; for a detailed exposition we refer to the original article by Zhou and Whitehead.^15^

#### Logistic model

**Figure 1.**
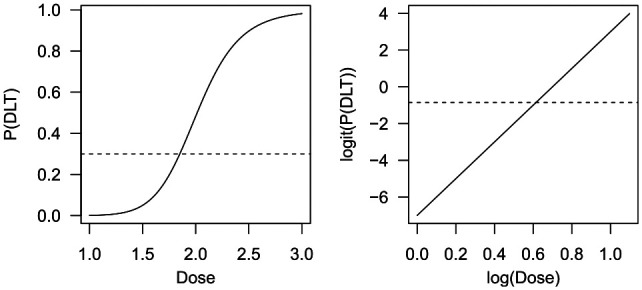
Example of an S-shaped dose-toxicity curve (left) and the corresponding straight line after transformation (right). The dotted horizontal line indates a target toxicity level of 0.3, or log(0.3/0.7) = –0.847 on the logit scale. P(DLT): probability of a dose-limiting toxicity.

We assume the relationship between dose and risk of toxicity follows a logistic model

$$\log\left(\frac{P(\mathrm{dose}-\mathrm{limiting}\;\mathrm{toxicity})}{1-P(\mathrm{dose}-\mathrm{limiting}\;\mathrm{toxicity})}\right)=\beta_{0}+\beta_{1}\log(\mathrm{dose})$$

where the logit transformation of the probability (i.e. the log odds) of observing a dose-limiting toxicity (left-hand side of the equation) is assumed to depend on the log-transformed dose in a linear fashion (right-hand side of the equation; see the illustration in Figure 1). We use toxicity data from study patients to estimate the values of the model parameters $\beta_{0}$ (intercept) and $\beta_{1}$ (slope).

#### Prior information

To get the Bayesian decision procedure started before any patient data are available, we need prior information on the dose-toxicity relationship. Guessing values of the model parameters $\beta_{0}$ and $\beta_{1}$ would be hard, so we prefer to formulate our prior beliefs about the toxicity rates of two distinct doses. The strength of these beliefs can be expressed in terms of their ‘effective sample sizes’:^27^ the information from, say, three pseudo-observations will be weighted as if they had been obtained from three real patients, and mathematically converted into so-called beta priors.^15^

#### Gain function

A gain function can be used to quantify, for each dose, the advantage of prescribing different dose levels to the next cohort of patients, where larger gains are to be preferred. Thus, the gain function helps to determine which dose should be recommended for the next patient cohort. The ‘patient gain’ function would assign the dose currently thought to be closest to the target toxicity level (which is optimal from a current patient’s perspective), whereas the ‘variance gain’ function would choose the dose that will likely maximise learning about the dose-toxicity relationship (which is optimal from an investigator’s perspective and also from the perspective of future patients who will be treated beyond the current clinical trial).^5^ In practice, the choice of gain function is unlikely to have a significant impact on the performance characteristics of the study design, but this can be explored in MoDEsT.

#### Escalation and stopping rules

Dose recommendations are determined primarily by the model and the gain function, but we may wish to apply additional restrictions such as:

- Always start at the lowest dose;
- Do not skip over any doses when escalating;
- Do not escalate upon observing a toxicity in the current cohort.

Stopping recruitment to the study will be recommended once

- The maximum number of patients have been analysed;
- A pre-defined maximum number of consecutive patients receiving the same dose has been reached;
- A sufficiently accurate estimate of the maximum tolerated dose has been obtained and/or;
- No dose among those in the prespecified set is deemed safe.

## Results

### The MoDEsT app

MoDEsT comes in two parts: a ‘Design’ module to investigate candidate design options and simulate their operating characteristics, and a ‘Conduct’ module to guide decision making throughout the study, incorporate accruing patient data into the model and provide summaries of the final dataset on completion of the study. Both modules are fully reactive, that is, changes made by the user become effective in real time. MoDEsT can be accessed online (https://medstats-lancs.shinyapps.io/design/ and https://medstats-lancs.shinyapps.io/conduct/) from any device with a web browser. For R users, the app is also available in the add-on package modest.^28^ We will keep maintaining both the web app and the R package (including bug fixes and possibly adding new options); hence, the appearance and functionality of MoDEsT may change slightly as it evolves.

#### The ‘Design’ module

The ‘Design’ module takes as inputs the basic study parameters (maximum sample size, cohort size, dose levels, target toxicity level, gain function), the pseudo-observations needed to specify prior distributions for parameters of the dose-toxicity model, ‘true’ values of model parameters for simulation of the Bayesian procedure in different scenarios, and additional escalation and stopping rules as detailed above; all these are conveniently specified via sliders, text boxes, and tick boxes (Figure 3). The app then creates graphical displays of the dose-toxicity curves, simulates an example of a study given the current specifications, and suggests a variety of scenarios^*^ for use in a subsequent simulation study (Figure 4). For the scenario chosen MoDEsT assesses a variety of operating characteristics and presents the results in tables and graphics. On the basis of the inputs the app generates a CSV design file that can subsequently be fed into the ‘Conduct’ module. In addition, a report summarising the design, prior information, and simulation results can be downloaded in PDF format.

#### The ‘Conduct’ module

The ‘Conduct’ module requires the user to upload a design file (obtained from the ‘Design’ module) and supply (anonymised) patient data. The latter can either be uploaded as a CSV file (typically created with a text editor or spreadsheet software such as Microsoft Excel, OpenOffice/LibreOffice Calc, or Google Sheets), or entered manually via a spreadsheet interface. The app then produces graphical displays of the data, fits the logistic model, calculates the current estimate of the maximum tolerated dose, and recommends either a dose for the next cohort or stopping the study in case a relevant criterion is fulfilled (Figure 5). A PDF report summarising the design, data, analysis, and recommendation is available for download. This can all be easily produced by the clinical study team for each dose review meeting so that dose recommendations based on statistical analyses of current and past patient data are used in real time alongside clinical opinion from the investigators to decide on dosing for the next patient cohort.

#### Getting help

While the app’s user guidance should be intuitive and most inputs and outputs self-explanatory, additional help may occasionally be required. The quickest way to learn more about an input element (e.g. slider, button, check box, text box) is by mousing over it, and a tooltip will appear. A full description/documentation of all of MoDEsT’s functionality along with a detailed explanation of all inputs and outputs is given in the help pages on the website. They are also included in the R package in the form of two HTML vignettes (https://cran.r-project.org/web/packages/modest/vignettes/Design.html and https://cran.r-project.org/web/packages/modest/vignettes/Conduct.html).

### Example: a phase I study of quercetin

The workflow when designing and conducting a study with MoDEsT is best illustrated with a real data example. In the following, we re-design and re-analyse (parts of) a dose-escalation study of a novel drug product in terminal cancer that originally used a 3 + 3-type design with several spontaneous modifications.

#### Dataset

Ferry *et al.* conducted a phase I study of the flavonoid quercetin in cancer patients suffering from a variety of forms of solid tumour no longer amenable to standard therapies.^30^ They assessed 9 dose levels (60, 120, 200, 300, 420, 630, 945, 1400, 1700 mg/m^2^) with the aim of finding the maximum tolerated dose under the premise that a 20% risk of renal toxicity (WHO grade 2) would be acceptable. A maximum of 18 patient cohorts of size 3 was to be recruited to the study.

**Figure 2.**
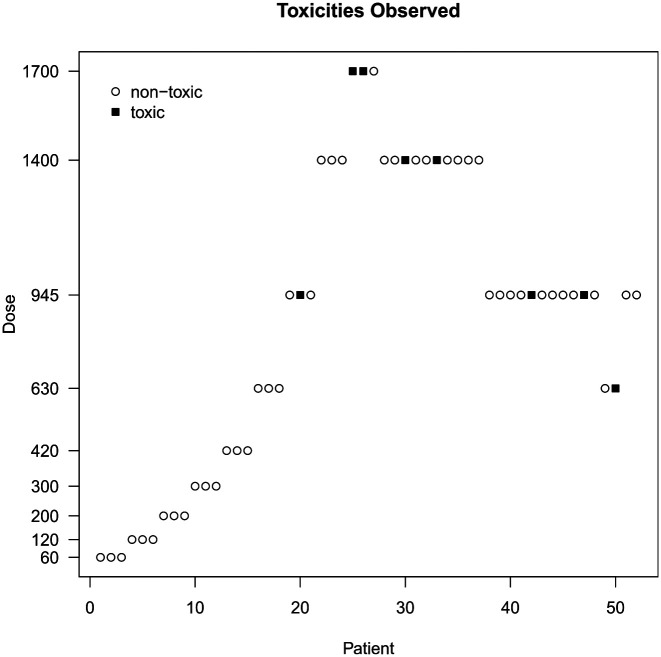
Overview of the quercetin study data.

Figure 2 provides an overview of the study data from a total of 52 patients. We note a number of oddities and decisions made against the rules of 3 + 3:

- The dose was escalated to 1400 mg/m^2^ for the 8th cohort despite a dose-limiting toxicity having been recorded for the 7th cohort at 945 mg/m^2^.
- Dose-limiting toxicities occurred in both the 10th and 11th cohort at 1400 mg/m^2^, and yet the dose was not de-escalated for the 12th cohort.
- No dose-limiting toxicities occurred in the 12th cohort, but the dose was de-escalated for the 13th cohort to 945 mg/m^2^.
- The 12th cohort consisted of four patients.
- A dose-limiting toxicity was recorded for the 17th cohort at 630 mg/m^2^, still the dose was escalated to 945 mg/m^2^ for the 18th cohort.
- The 16th, 17th, and 18th cohort each consisted of two patients only.

These deviations cannot be incorporated within the 3+3 design with its inherent inflexibility. This design no longer provides a relevant contribution to dose-escalation decisions, whereas the model-based procedure implemented in MoDEsT can easily handle cohorts of non-standard size and dose recommendations overruled by clinical judgement.

#### Study design

**Figure 3.**
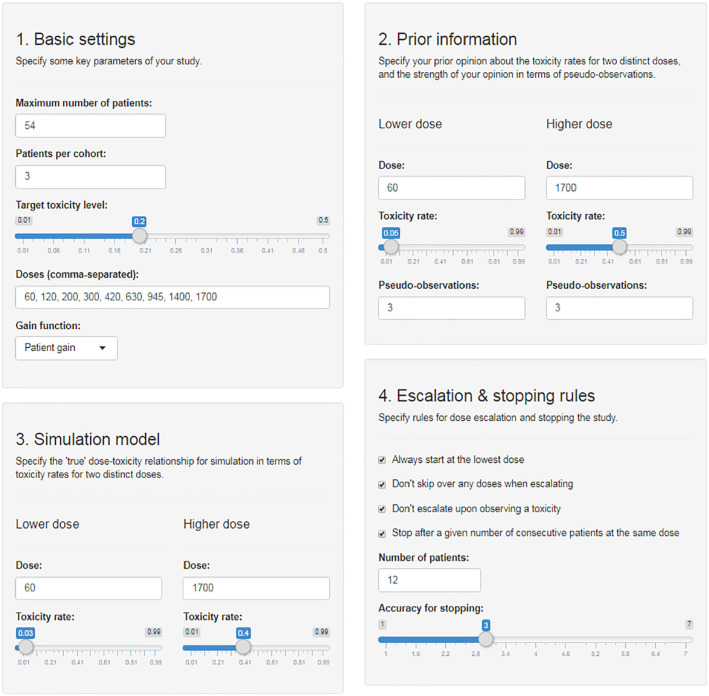
Input mask of the ‘Design’ module with specifications inspired by the quercetin study.

We set the study design parameters (maximum sample size, cohort size, dose levels, target toxicity level) as in Ferry *et al.’*s original study and use the patient gain function to drive dose-escalation recommendations (Figure 3). We specify a ‘pessimistic’ or ‘conservative’ prior distribution for parameters of the dose-toxicity relationship by specifying pseudo-observations consistent with the opinion that, a priori, we would expect to see 0.3 dose-limiting toxicities if three patients were treated with 60 mg/m^2^ and 1.5 dose-limiting toxicities if three patients received 1700 mg/m^2^, corresponding to anticipated dose-limiting toxicity risks of 10% and 50%, respectively. Note that non-integer values of dose-limiting toxicities are acceptable when specifying prior distributions. For the assumed ‘true’ dose-toxicity model used to simulate dose-limiting toxicity occurrences we choose the ‘true’ dose-limiting toxicity risks on the lowest and highest doses to be 3% and 40%, respectively. One does not necessarily have to specify priors by considering dose-limiting toxicity risks on the lowest and highest dose levels, but we do recommend choosing one dose at the lower and one at the upper end of the spectrum.

For the purpose of this example, we enforce starting at the lowest dose, not skipping over any doses when escalating, and not escalating when a dose-limiting toxicity occurs in the current cohort. We consider trial designs which would recommend stopping the study for accuracy once 12 consecutive patients have received the same dose, or when the ratio of the upper and lower 95% credible limit around the estimated maximum tolerated dose is 3 or less.

**Figure 4.**
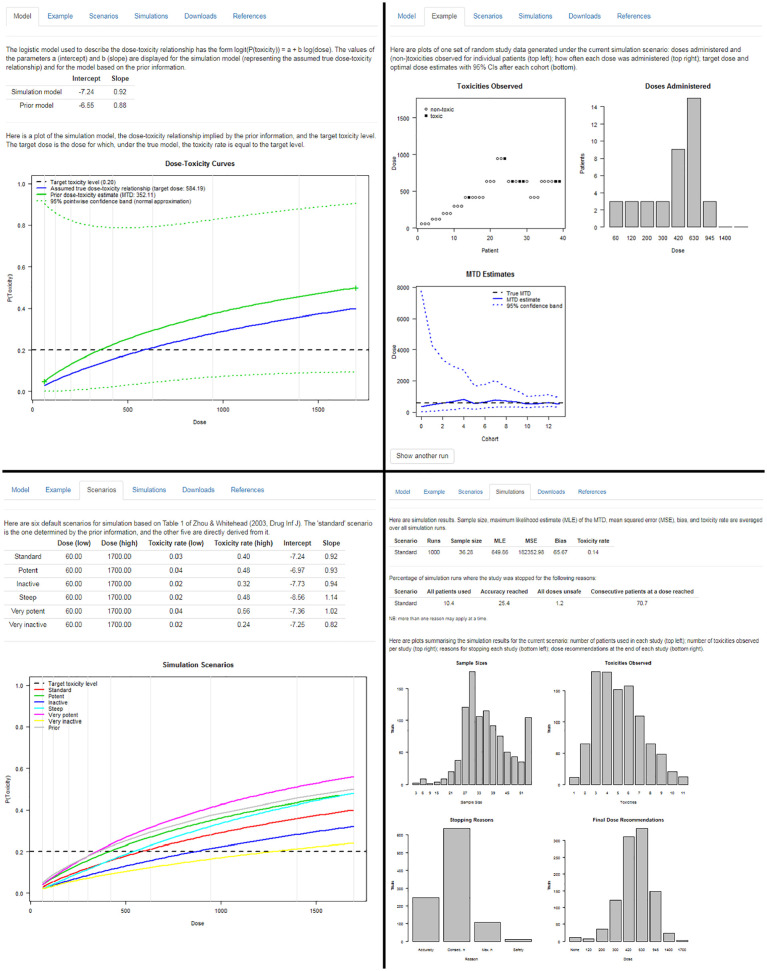
Top left: ‘Model’ tab of the ‘Design’ module displaying the prior and the assumed true dose-toxicity curve. Top right: ‘Example’ tab of the ‘Design’ module showing one simulated example dataset. Bottom left: ‘Scenarios’ tab of the ‘Design’ module giving an overview of six simulation scenarios. Bottom right: ‘Simulations’ tab of the ‘Design’ module summarising simulation results.

From these inputs, MoDEsT generates a number of graphs and tables to summarise the operating characteristics of the stipulated design. We see that if our simulation model were indeed the true dose-toxicity curve, the maximum tolerated dose would be estimated as 584 mg/m^2^, but only 352 mg/m^2^ under the much more cautious prior model (Figure 4, top left panel). Unsurprisingly, the 95% credible band around the prior curve is extremely wide, as it is based on only three (pseudo-)observations.

MoDEsT displays one simulated realisation of a study which proceeds according to the proposed Bayesian dose-escalation procedure and stopping rules, simulating patient outcomes setting dose-limiting toxicity risks equal to values consistent with the current dose-toxicity simulation model. This is intended as an illustrative example of what the study *could* look like. A single simulated dataset, and the corresponding evolution of the dose-escalation trial, will not necessarily be representative of what would typically be observed if the true underlying dose-toxicity relationship was identical to the simulation model. However, by simulating a large number of trials and averaging across them, we can deduce what might happen on average. MoDEsT is designed to allow easy repeated simulation, which will be helpful in getting a feel for the variation of output that can occur.

The example trial shown in Figure 4 (top right panel) is stopped after 13 cohorts, when a sufficiently accurate estimate has been obtained in accordance with the pre-specified stopping rules. The 95% credible band for the maximum tolerated dose becomes narrower over the course of the study and always contains the ‘true’ value of 584 mg/m^2^.

To facilitate assessment of the design’s operating characteristics, MoDEsT automatically creates six simulation scenarios that can be used in the simulation study: the standard scenario defined by the ‘true’ simulation model, and five additional scenarios derived from it that imply lower (‘inactive’) or higher (‘potent’) toxicity rates over the whole dose range or parts of it. They are summarised in a table and graph, alongside the prior for comparison (Figure 4, bottom left panel).

Figure 4 (bottom right panel) shows summary tables and graphs of 1000 simulations performed within seconds by the press of a button under the (anticipated) standard scenario. In this example, the average sample size required was 36.28 patients (averaged over all 1000 simulated trials), the average of the maximum likelihood estimate of the maximum tolerated dose was 649.86 mg/m^2^, with a large mean squared error and notable bias, and an average toxicity rate of 14%, which is well below the targeted 20%. We see how many simulated trials were stopped for which reason(s); the sum of the percentages is greater than 100% because multiple stopping criteria can be fulfilled at the same time. We also get an overview of the sample sizes used, numbers of dose-limiting toxicities observed, reasons for stopping, and doses recommended as the maximum tolerated dose across the 1000 simulated trials. These summary plots and tables produced instantly by MoDEsT will be sufficient for most users but a detailed account of all individual simulation runs can be downloaded as a CSV file.

#### Study conduct

For illustrative purposes we present and discuss here only the analyses following the 7th and the last cohort, respectively; in practice a similar analysis would be performed after every single patient cohort.

We upload the design file and a CSV containing the study data. MoDEsT generates tabular overviews of the design parameters and patient data, as well as plots such as the one in Figure 2. We recommend that users review this output to double-check the information and also whether data have been read in as intended, that is, correct columns were specified for the cohort, dose, and response variable.

**Figure 5.**
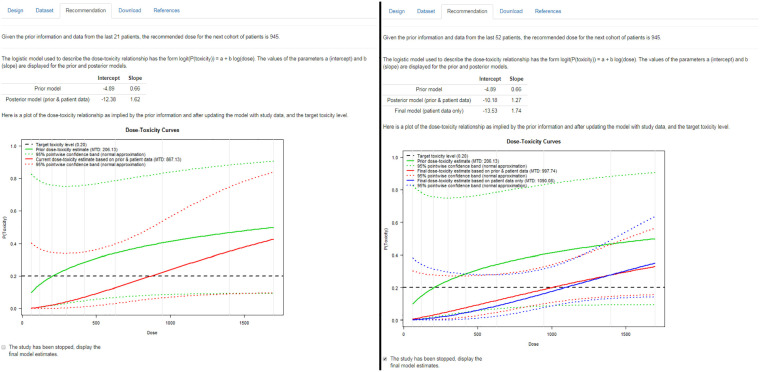
‘Recommendation’ tab of the ‘Conduct’ module after the 7th cohort (left) and after the final analysis (right) of the quercetin study.

In Ferry *et al.’*s study, the dose was escalated from 945 to 1400 mg/m^2^ for the 8th cohort, despite a dose-limiting toxicity being observed in the 7th cohort (Figure 2). Re-analysing the data up to and including the 7th cohort with MoDEsT, we find that the Bayesian dose-escalation procedure recommends administering 945 mg/m^2^ to the 8th patient cohort (Figure 5, left panel), which is in line with the stipulated safety rule of not escalating when a dose-limiting toxicity has been observed in the current cohort. Due to the amount of data accruing, the 95% credible band is much narrower than for the prior.

The dataset accrued upon completion of the study comprises 52 patients, which is less than the envisaged maximum sample size of 54, so we have to tick the box in MoDEsT to indicate that the study has been stopped (Figure 5, right panel). The final model-based estimate of the maximum tolerated dose is 998 mg/m^2^, but this is influenced by the very pessimistic prior. Removing the pseudo-observations used to formulate the prior yields a final (maximum likelihood) estimate of 1090 mg/m^2^ and a marginally wider credible band. Both estimates lead to a recommendation of 945 mg/m^2^ for the maximum tolerated dose, which is the same as in Ferry *et al.* However, had MoDEsT been used in the original study, the deviations from the protocol (such as smaller and larger cohorts) could easily have been accommodated, and the (clearly too high) dose of 1700 mg/m^2^ would probably never^†^ have been administered.

## Discussion

Building trust in the utility, safety, and practical applicability of model-based dose-escalation designs is an essential step towards their wider acceptance within the clinical community. To assist this process, we have created MoDEsT, a software tool that is straightforward to use even without any specialist knowledge of statistical programming. We are positive it will convince trialists and statisticians that model-based methods are a feasible and worthwhile alternative to the 3 + 3 design and can be implemented with limited additional effort. Tasks that currently discourage many clinicians from using model-based designs, like having to set a prior, are made simple in MoDEsT. One of the main advantages of the software is that it allows investigators to input different (hypothetical or real) datasets ahead of time to see what dose recommendations the Bayesian dose-escalation procedure would generate, allowing them to develop some intuition as to how the procedure is working and how it would compare with their own intuition or algorithmic rules they might be more familiar with. We hope that by seeing the effects of changing design parameters in real time, trialists will become more confident in using model-based designs and that these will increasingly become the norm in early-phase dose-escalation studies.

## Notes

* The simulation scenarios are based on Table 1 of Zhou & Whitehead (2003)^15^ and Table 7.9 of Whitehead (2006).^29^

† Simulations with the final model of Figure 5 (right panel) as the ‘true’ simulation model show that it is extremely unlikely to ever visit this dose level.
